# Modified Baihu decoction therapeutically remodels gut microbiota to inhibit acute gouty arthritis

**DOI:** 10.3389/fphys.2022.1023453

**Published:** 2022-12-15

**Authors:** Xianyang Wang, Haishan Long, Ming Chen, Zongbo Zhou, Qinlin Wu, Shijie Xu, Geng Li, Zhifu Lu

**Affiliations:** ^1^ Animal Experiment Center, Guangzhou University of Chinese Medicine, Guangzhou, China; ^2^ Haikou Hospital of Traditional Chinese Medicine, Haikou, Hainan, China; ^3^ Guangzhou University of Chinese Medicine, Guangzhou, China

**Keywords:** Baihu decoction, acute gouty arthritis, gut microbiota, proinflammatory cytokines, traditional medicine

## Abstract

**Background:** Acute gouty arthritis (AGA) is the most common first symptom of gout, and the development of gout as a metabolic and immune inflammatory disease is also correlated with the gut microbiota. However, the mechanism of the effect of changes in the gut microbiota on AGA remains unclear. The intestinal flora can not only affect purine metabolism or regulate inflammation, but also influence the therapeutic effect of drugs on AGA. The aim of this study was to investigate the exact mechanism of modified Baihu decoction (MBD) in the treatment of AGA and whether it is related to the regulation of the structure of the intestinal flora.

**Methods:** On the 21st day of MBD administration by continuous gavage, a rat acute gouty arthritis model was constructed using sodium urate (0.1 mL/rat, 50 mg/mL), and the ankle joint swelling was measured before and 4 h, 8 h, 24 h, and 48 h after the injection of sodium urate. After 48 h of sodium urate injection, serum, liver, kidney, ankle synovial tissue and feces were collected from rats. The collected samples were examined and analyzed using H&E, Elisa, Immunohistochemistry, Histopathology, 16S rDNA, and Biochemical analysis. To investigate the mechanism of MBD to alleviate AGA using pro-inflammatory factors and intestinal flora.

**Results:** MBD (5.84, 35 g/kg) was administered orally to AGA rats and diclofenac sodium tablets (DS-tablets) were used as standard treatment control. Serum biochemical assessment confirmed that MBD is a safe drug for the treatment of AGA. In addition, our findings confirmed that MBD relieved AGA-related symptoms, such as toe swelling. Lowering serum levels of uric acid, IL-1β, and TGF-β1 immunohistochemical results also confirmed that MBD reduced the expression of inflammatory elements such as IL-1β, NLRP3, ASC, and Caspase-1 in synovial tissue.Furthermore, compared with control group, the 16s rDNA sequencing of AGA rat faeces revealed an increase in the relative abundance of *Lachnospiraceae, Muribaculaceae, and Bifidobacteriaceae* species. While the relative abundance of *Lactobacillaceae, Erysipelotrichaceae, Ruminococcaceae, Prevotellaceae and Enterobacteriaceae* showed a relative decrease in species abundance. Of these, the reduction in species abundance of *Enterobacteriaceae* was associated with a reduction in amino acid metabolism and environmental perception. After MBD therapeutic intervention, the disturbance of the intestinal flora caused by AGA was restored.

**Conclusion:** In summary, MBD is an effective agent for the treatment of AGA, with the potential mechanism being the regulation of intestinal flora to control inflammation. This would help to promote the therapeutic effect of MBD on AGA.

## Introduction

As the standard of living improves, the recurrence and incidence rates of gout continue to rise due to increased consumption of cholesterol and purine-rich foods such as meat and seafood (Batt et al., 2014). Global gout prevalence climbed from 0.1% in 2010 to over 10% by 2020 (Afinogenova et al., 2022; Huddleston and Gaffo 2022), and the prevalence of gout in China has increased to 1–3% (Keller and Mandell 2021). The affected population is characterized by a young age, and the disease is closely associated with the development of obesity, hypertension, coronary heart disease, hyperlipidaemia, atherosclerosis, diabetes mellitus, and insulin resistance (Smith et al., 2014; Wilson and Saseen 2016; Ragab et al., 2017). Gout is an inflammatory arthropathy caused by purine metabolism or uricosuric disorders and clinically manifested by acute recurrent arthritis, chronic arthritis, tophaceous gout, renal calculi, and urate nephropathy (Dalbeth et al., 2021). In particular, AGA has a predilection for the lower limb joints and appears as a rapidly progressing severe disorder with an abrupt onset, which reaches peak intensity within 6 h and undermines the quality of life of AGA patients as they suffer from swelling, thermalgia, and severe pain in the affected joint and its surrounding soft tissue (Tan et al., 1993).

Recent studies have demonstrated that AGA usually occurs when the articular cavity is oversaturated with urate crystals that elicit an inflammatory response (Wilson and Saseen 2016). Because primary gout is now not curable, clinical medication for the condition mainly aims to suppress uric acid (UA) synthesis, promote UA excretion and control its sudden onset. The adverse effects (e.g., gastrointestinal bleeding) on the body are caused by the combination of drugs such as colchicine, non-steroidal anti-inflammatory drugs (NSAIDs) and glucocorticoids (GCs) (Kogut et al., 2020). Therefore, the discovery of herbs for the treatment of AGA through the study of herbs has become a research hotspot.

In *Shanghan Lun* (also known as the *Treatise on Cold Damage Diseases*), Baihu decoction is a classic prescription composed of gypsum, Anemarrhena asphodeloides Bunge, Glycyrrhiza inflata Batalin, and polished round-grained rice. In modern medicine, Baihu decoction is useful to relieve fever, reduce inflammation and strengthen the immune system in patients with febrile conditions, rheumatoid arthritis (RA), and systemic inflammation (Zhang et al., 2013; He et al., 2019; Liu et al., 2021). A study by Le Shi et al. (Shi et al., 2021) found that Achyranthis bidentatae Radix and *Lonicera japonica* Thunb, which are consistent with the herbs in MBD, protected rats from monosodium urate-induced experimental gouty arthritis. We therefore think that MBD might have a similar effect on AGA.

Recently, ample evidence has demonstrated the dynamic interaction between the gut microbiota and the host innate and adaptive immune systems (Yoo et al., 2020). Gut dysbiosis can adversely affect the host immune response and accelerate the development of diverse inflammatory diseases, which also occur as a result of AGA-induced inflammation (Nieuwdorp et al., 2014). For instance, *Prevotella*, a bacterial species isolated from the gut microbiota in patients with RA, is reported to inhibit the development of arthritic problems (Rowland et al., 2018). In addition, how the gut microbiota affects UA metabolism has become an emerging research topic (Mendez-Salazar and Martinez-Nava 2022). As an independent marker or risk factor, UA is the end-product of endogenous purine metabolism, of which the conversion involves a series of enzymes that may lead to abnormal UA metabolism (Rowland et al., 2018; Mendez-Salazar et al., 2021). In addition to UA production and excretion, certain endogenous microorganisms are recognized as key players in UA metabolism (Choi and Song 2016; Pan et al., 2020). Compared with healthy individuals, hyperuricaemia (HUA) and gout patients experience differential changes in the gut microbiota (Zhang et al., 2013; Forbes et al., 2016; Kondratiuk et al., 2020). UA-lowering treatment can alter the gut microbiota composition, and probiotics seem antagonistic to abnormal UA metabolism (Kedia et al., 2021; Su et al., 2021). Therefore, it is expected to help identify molecular targets for the diagnosis and treatment of AGA by studying the interaction between the gut microbiota and UA metabolism and its effect on AGA. This study aims to show that intestinal flora plays a key role in the development of disease in AGA.

In this study, the AGA model was constructed using sodium urate and treated with MBD. MBD was given *via* gastric lavage to evaluate the efficacy and safety of MBD on AGA rats and to analyse the effect of MBD on the intestinal flora of AGA rats (Morrison and Preston 2016; Yun et al., 2017). Through the above experiments, this paper will elucidate the specific mechanism of MBD for the treatment of AGA by regulating intestinal flora and provide a scientific basis for MBD for the treatment of AGA.

## Materials and methods

### Animals

Male Sprague-Dawley (SD) rats, weighing 200–220 g, aged 6 weeks were purchased from Guangdong Medical Laboratory Animal Center (Certificate No. SCXK (Guangdong) 2013-0002). Five rats were kept in each cage, and they were housed in an environment at 22±1°C with 40% humidity and a 12 h photoperiod. All procedures are ethical for animal testing and approved by the Guangzhou Forevergen Biosciences Application for Laboratory Animal Welfare and Ethical review. IACUC Issue NO: IACUC-AEWC-F2112011.

The AGA group, which received the same amount of clean water by gavage coupled with each feeding of a high-fat meal and honey water (200 g/L), and the control group, which had a usual diet and drinking water, were divided into two groups of animals. The high-fat diet, honey water (200 g/L), and MBD gavage were given daily to the low MBD group (5.84 g/kg/d) and the high MBD group (35 g/kg/d). Along with the high-fat diet, the DS-pill group also received honey water (200 g/L) and diclofenac sodium extended-release pills (13.5 mg/kg/d) daily. We used sodium urate to create an AGA rat model on the 21st day of treatment. The right ankle joint of the rats was injected with sodium urate (0.1 mL/rat, 50 mg/ mL) and 1% sodium pentobarbital to generate acute gouty arthritis (Lin et al., 2020; Zhang et al., 2021). A similar dose of sodium chloride (0.9% concentration) was injected into the control group in the same spot.

### Chemicals and Reagents

Uric acid sodium salt used to be bought from Sigma, batch quantity BCCB4889. Sodium dichlorophenolate sustained-release capsules have been bought from Novartis Pharmaceutical Co., Ltd., with the countrywide drug preferred of H10980297. Rat Interleukin 1β (IL-1β) ELISA Kit, Rat Tumor Necrosis Factor α (TNF-α) ELISA Kit, Rat Transforming Growth Factor β1 (TGF-β1) ELISA Kit had been bought from Wuhan Purity Biotechnology Co., Ltd. To kind organic obvious agent was once bought from Guangxi Cenxi Rosin Factory. Hematoxylin staining answer (Batch No. 181537) and eosin staining answer (Batch No. XH185207) have been bought from Wuhan Saiweier Biological Technology Co., Ltd. Ready-to-use UItraSensitive s-p hypersensitive package (rabbit), DAB coloration package bought from Maixin Biotechnology Co., Ltd. TNF-α antibody and IL-1β antibody had been bought from ABclonal Technology. NLRP3, ASC and Caspase-1 antibodies have been bought from Proteintech. High purity complete RNA fast extraction package (centrifugal column type) used to be bought from Beijing BioTeke Biotechnology Co., Ltd. HiScript II Q RT SuperMix for qPCR (+gDNA wiper).

### Preparation and HPLC analysis of MBD

MBD consists of gypsum (120 g), Anemarrhena asphodeloides Bunge (20 g), Paeonia lactiflora Pall (20 g), Achyranthis bidentatae Radix (15 g), Corydalis yanhusuo (20 g), Phellodendron amurense Rupr. (15 g), Atractylodes lancea (30 g), Glycyrrhiza inflata Batalin (10 g), Coix lacryma-jobi L (30 g), *Lonicera japonica* Thunb (30 g), Reynoutria japonica Houtt (20 g), and Clematis chinensis Osbeck (20 g). All crude herbs were provided by Xing Yuan Chun Pharmacy in Guangzhou and licenced by Associate Professor Liangwen Yu. A packet of Chinese natural remedy (350 g) was soaked in 1 L of distilled water for 30 min and decocted in boiling water for 3 h. The solution was eliminated, and the residue was removed. The final solution was heated and targeted to 100 ml for the high concentration drug dose (3.5 g/ml). Then, 10 ml of the high-dose formula was taken, and 50 ml of distilled water was added for dilution to a low dose (0.584 g/ml). Raw substances were saved at room temperature. MBD preparations were saved at −40°C.

HPLC-MBD evaluation was carried out at 220 nm for protocatechuic aldehyde, chlorogenic acid, puerarin, caffeic acid, paeoniflorin, polydatin, luteolin, quercetin, and naringenin using a Thermo Scientific U3000 HPLC system. Chromatographic separation was accomplished using a Phenomenon®Luna-C18 analytical column (4.6 mm × 250 mm, 5 μm). Protocatechuic aldehyde (lot numbers: 110810-201007, purity: 98.20%, CAS: 139-85-5), chlorogenic acid (lot numbers: 140687-201503, purity: 99.90%, CAS: 327-97-9), puerarin (lot numbers: HP148456198, purity: 98.0%, CAS: 3681-99-0), caffeic acid (lot numbers: 110885-200102, purity: 98.20%, CAS: 331-39-5), paeoniflorin (lot numbers: 110736-201337, purity: 94.90%, CAS: 23180-57-6), polydatin (lot numbers: ZM0530LA14, purity: 98.0%, CAS: 65914-17-2), luteolin (lot numbers: HL090610298, purity: 98.0%, CAS: 491-70-3), quercetin (lot numbers: 05-2013, purity: 98.0%, CAS: 117-39-5), and naringenin (lot numbers: HN206559198, purity: 98.0%, CAS: 67604-48-2) were from China Academy of Food and Drug Testing and Certification. Ten millilitres of the decocted and filtered solution of MBD in a water bath were evaporated and dried. The samples were filtered *via* a microporous membrane. Chromatographic conditions: Phenomenon Luna C18 2) 100A (4.6 mm × 250 mm, 5 µm). Acetonitrile (A) 0.1% phosphoric acid (B) was the amobile phase. The flow charge used was 1 ml/min, and the column temperature was 25 °C. The detection wavelength used was 220 nm. The injection volume of the standard was 10 μL or 5 μL, and the injection volume of the sample for the test was 20 μl.

### Toe swelling evaluation

Toe swelling was measured by using a Vernier calliper before and 0, 4, 8, 24, and 48 h after uric acid sodium salt injection to consider the incidence of arthritis. The swelling was calculated using the following formula: toe swelling diploma (mm) = b-a, where the place b is the proper hind paw swelling diploma after inflammation, and a is the proper hind paw swelling diploma before irritation (Wu et al., 2018).

### Histopathology

Ankle synovial tissue, kidney, and liver tissue sections were fixed with 4% paraformaldehyde solution overnight, dehydrated, embedded in paraffin blocks and sectioned at 3 µm. The sections were further deparaffinized and hydrated and then stained with haematoxylin-eosin (H&E). The staining results were observed under a positive phase contrast microscope.

### Biochemical analysis

After blood collection, blood samples were left to stand for a single day at 4 °C and centrifuged at 3000 × g for 15 min. The supernatant was taken for biochemical detection with a Roche biochemical analyser. The levels of creatinine (Cr), UA, total protein (TP), albumin (ALB), C-reactive protein (CRP), aspartate aminotransferase (AST), and alanine aminotransferase (ALT) in serum were detected.

### Elisa

After ankle joint injection of uric acid sodium salt for 48 h, the rats were anaesthetized *via* intraperitoneal injection, blood was collected from the stomach aorta, and serum was separated. The levels of IL-1β, TNF-α, and TGF-β1 in the serum of rats were detected using the appropriate test kit according to the manufacturer’s instructions (Wuhan Pure Biotechnology Co., Ltd.).

### Immunohistochemistry

The expression of TNF-α, IL-1β, NLRP3, ASC, and Caspase-1 in synovial tissue was detected by the immunohistochemical SP method. The synovial tissue was transparently immersed in 58–60°C paraffin with the aid of gradient alcohol and dehydrated in xylene for embedding; paraffin blocks were sectioned into 3-μm slices with the aid of a slicer, and then the slices were displayed in a 42°C water bath. After baking at 60°C, the slices were incubated for 1 day at 37°C. Then, dewaxing, gradient ethanol hydration, and high-temperature repair were performed. The tissues were circled with an immunohistochemical pen and incubated with PBS for 15 min. Each area was treated with 50 μl peroxidase blockading answer (reagent A) to block the endogenous peroxidase activity, incubated at room temperature for 15 min, and washed with PBS 3 times for 3 min each time. Each area was brought with 50 μL of non-immune animal serum (reagent B) and incubated at room temperature for 30 min. Each area received 50 μL primary antibody working solution (TNF-α, IL-1β, NLRP3, ASC dilution ratio used to be 1:200, Caspase-1 dilution ratio used to be 1: 400) and incubated at 4°C. Slices were rinsed three times for 10 min each in PBS. Each slice was treated with 50 μl of biotin-labelled secondary antibody (reagent C), incubated at room temperature for 40 min, and washed with PBS three times for 3 min each time. Each slice was treated with 50 μl streptavidin-peroxidase answer (reagent D), incubated at room temperature for 15 min, and washed with PBS 3 times for 3 min each time. Then, 100 μl freshly organized DAB solution was added to every area and the slices were visualized under a microscope.

### Sequencing and analysis of 16S rDNA of faecal samples

The rat droppings were collected 1 hour prior to execution. The sequencing was carried out *via* Guangzhou Genedenovo Biotechnology Co., Ltd. Qualified DNA was once amplified with broad-range bacterial primers (341F: 5′-CCTACGGGNGGCWGCAG-3′ and 806R: 5′-GGACTACHVGGGTWTCTAAT-3′) concentrated on the V3-V4 location of the 16S rRNA gene. After genomic DNA was extracted from the samples, the conserved area of rDNA was amplified with particular primers with barcodes, and then the PCR amplification product was recovered *via* gel cutting and quantified using a QuantiFluorTM fluorescence spectrometer. The purified amplification products were blended in equal amounts, sequencing connectors were ligated, sequencing libraries were constructed, and Illumina PE250 was used for sequencing. After the raw reads were sequenced, we first filtered the low quality reads using FASTP software, then assembled them, spliced the double-ended reads into tags using FLASH software, and then filtered the tags to reap the statistics known as Clean tag. Next, we used the UPARSE algorithm of USEARCH software program to operate clustering primarily based on Clean Next. The clustering was carried out primarily based on Clean tag using USEARCH software’s UCHIME algorithm to eliminate the chimeric tag detected throughout the clustering comparison, and the final record obtained was the effective tag. After acquiring OTUs, OTU abundance records were carried out primarily based on the effective tag.

## Results

### HPLC analysis of modified Baihu decoction

**FIGURE 1:**
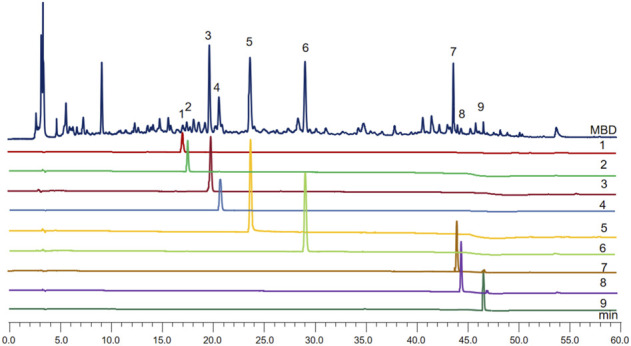
MBD chromatogram obtained by filtration of water extraction, evaporation in a water bath and dissolution in methanol. The *X*-axis is the retention time, and the *Y*-axis is the absorbance unit. 1. Protocatechuic aldehyde, 2. Chlorogenic acid, 3. Puerarin, 4. Caffeic acid, 5. Paeoniflorin, 6. Polydatin, 7. Luteolin, 8. Quercetin, and 9. Naringenin.

Because of the anti-inflammatory and uric acid-lowering consequences of MBD, HPLC was used once to screen the components accountable for the identified benefits. A total of nine advantageous compounds were detected in crude MBD extracts (Figure 1). The contents of protocatechuic aldehyde, chlorogenic acid, puerarin, caffeic acid, paeoniflorin, polydatin, luteolin, quercetin, and naringenin in MBD were 32.98 μg/g, 5.70 μg/g, 450.66 μg/g, 93.86 μg/g, 304.33 μg/g, 415.69 μg/g, 192.95 μg/g, 14.10 μg/g, and 27.94 μg/g, respectively (Supplementary Datasheet S1). Various principal elements in MBD, consisting of puerarin, paeoniflorin, polydatin, and luteolin, have anti-inflammatory properties.

### Effect of MBD on crystalline foot swelling with uric acid sodium salt

**FIGURE 2:**
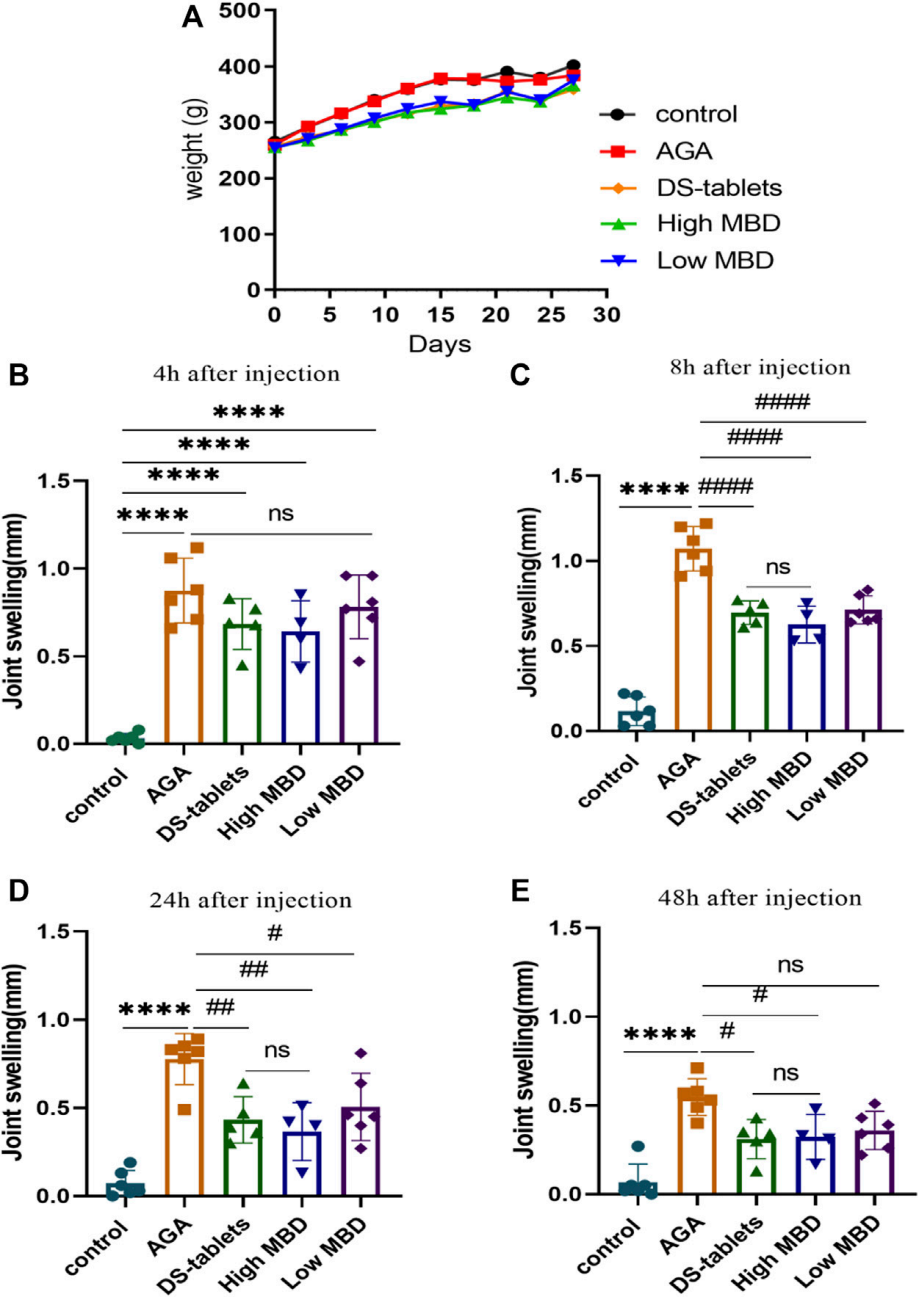
Effect of MBD on body weight and ankle swelling in AGA rats. **(A)** Changes in body weight. **(B)** Change in ankle swelling 4 h after injection. **(C)** Change in ankle swelling 8 h after injection. **(D)** Change in ankle swelling 24 h after injection. **(E)** Change in ankle swelling 48 h after injection. *p < 0.05, **p < 0.01, ***p < 0.001 indicated that the difference was statistically significant compared with the control group; *#p < 0.05,* ^*# #*^ *p < 0.01, #* ^*# # #*^ *p < 0.001* compared with the AGA group, the difference was statistically significant. N = 6/group.

As severe pain and swelling of the joints and periarticular areas are the most common symptoms of gout, we first investigated whether MBD ameliorated uric acid sodium salt-induced joint swelling. The body weight of the rats showed an increasing trend throughout the experimental cycle (Figure 2A). There was no significant difference in toe swelling between the groups prior to the injection of sodium urate. Four hours after the injection of sodium urate into the ankle joint of rats, the toes of rats in all groups except the control group were significantly swollen (Figure 2B), and the swelling peaked 8 h after the injection (Figure 2C). Toe swelling decreased with time after 8 h (Figure 2D). Compared with the AGA group, the MBD high- and low-dose groups had obvious effects on relieving toe swelling in rats. In addition, the DS-table group could significantly reduce the swelling of rat toes. There was no significant difference between MBD and DS tablets in reducing toe swelling (Figure 2E).

### Pathological effects of MBD on sodium urate-induced synovial tissue and liver and kidney

**FIGURE 3:**
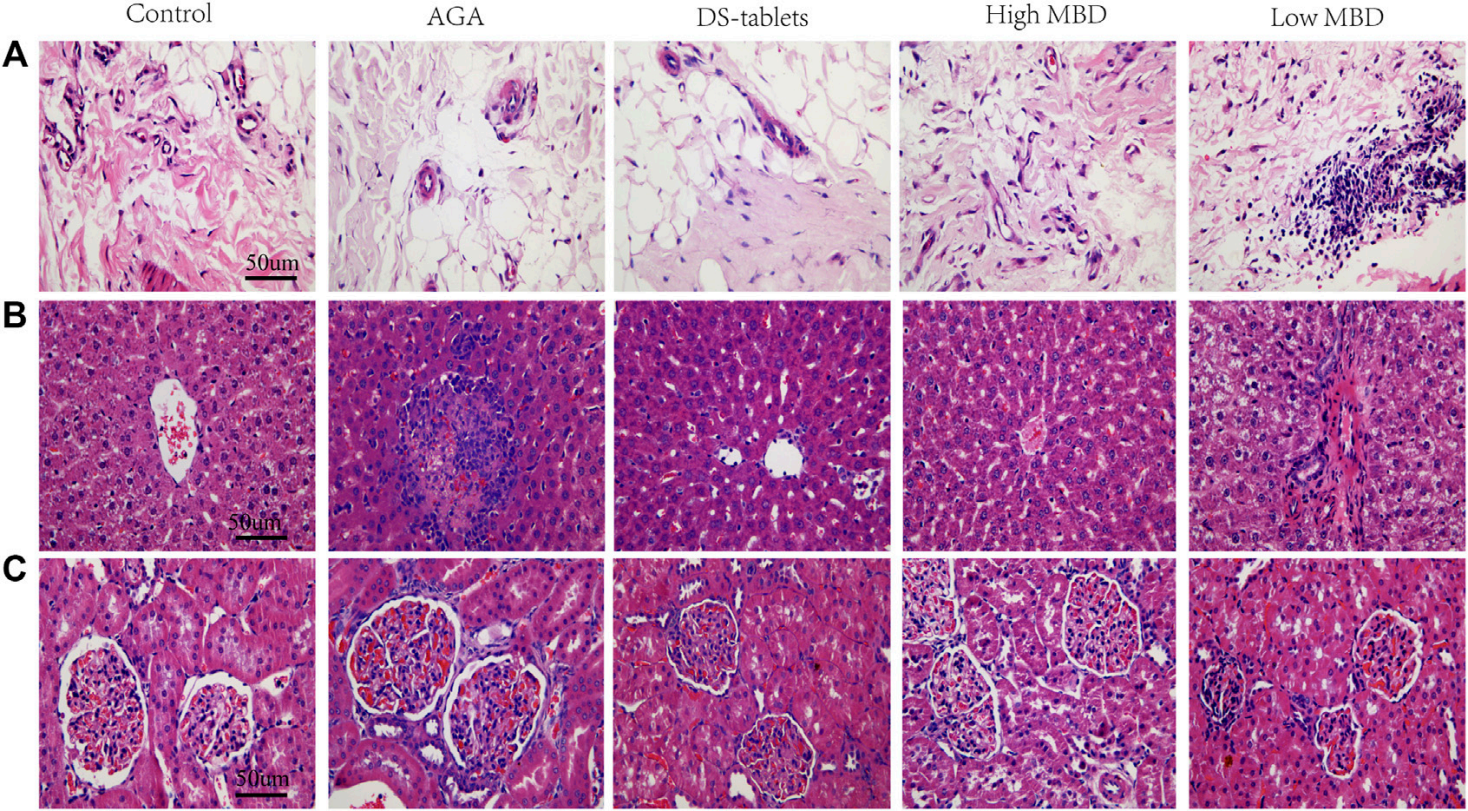
Pathological effects of MBD on sodium urate-induced synovial tissue and liver and kidney. **(A)** Synovial tissue. **(B)** Liver. **(C)** Kidney. Positive phase contrast microscope, magnification 400x.

In the synovial tissue, the AGA group showed proliferation of synovial cells, formation of multiple vascular opacities, interstitial oedema, and multiple inflammatory cell infiltrations. High doses of MBD effectively improved synovial cell proliferation and reduced vascular opacities and interstitial oedema (Figure 3A). In liver tissue, hepatocytes in the AGA group showed large patches of coagulative necrosis, with numerous inflammatory cell infiltrates and fibroblast proliferation. In the High MBD group, the hepatocytes were arranged radially along the central vein with a small amount of localized focal accumulation of inflammatory cells, while in the Low MBD group, the necrotic area of hepatocytes was located around the central vein with localized infiltration of inflammatory cells (Figure 3B). In the kidney tissue, there was a large amount of inflammatory cells infiltrating around the glomerulus and a small amount of focal accumulation of inflammatory cells and fibroblast proliferation in the tubules of the AGA group, while there was a small amount of inflammatory cells infiltrating around the glomerulus and a small amount of focal accumulation of inflammatory cells in the tubules of the High MBD group (Figure 3C).

### Effect of MBD on liver and kidney functions in rats with gouty arthritis

**FIGURE 4:**
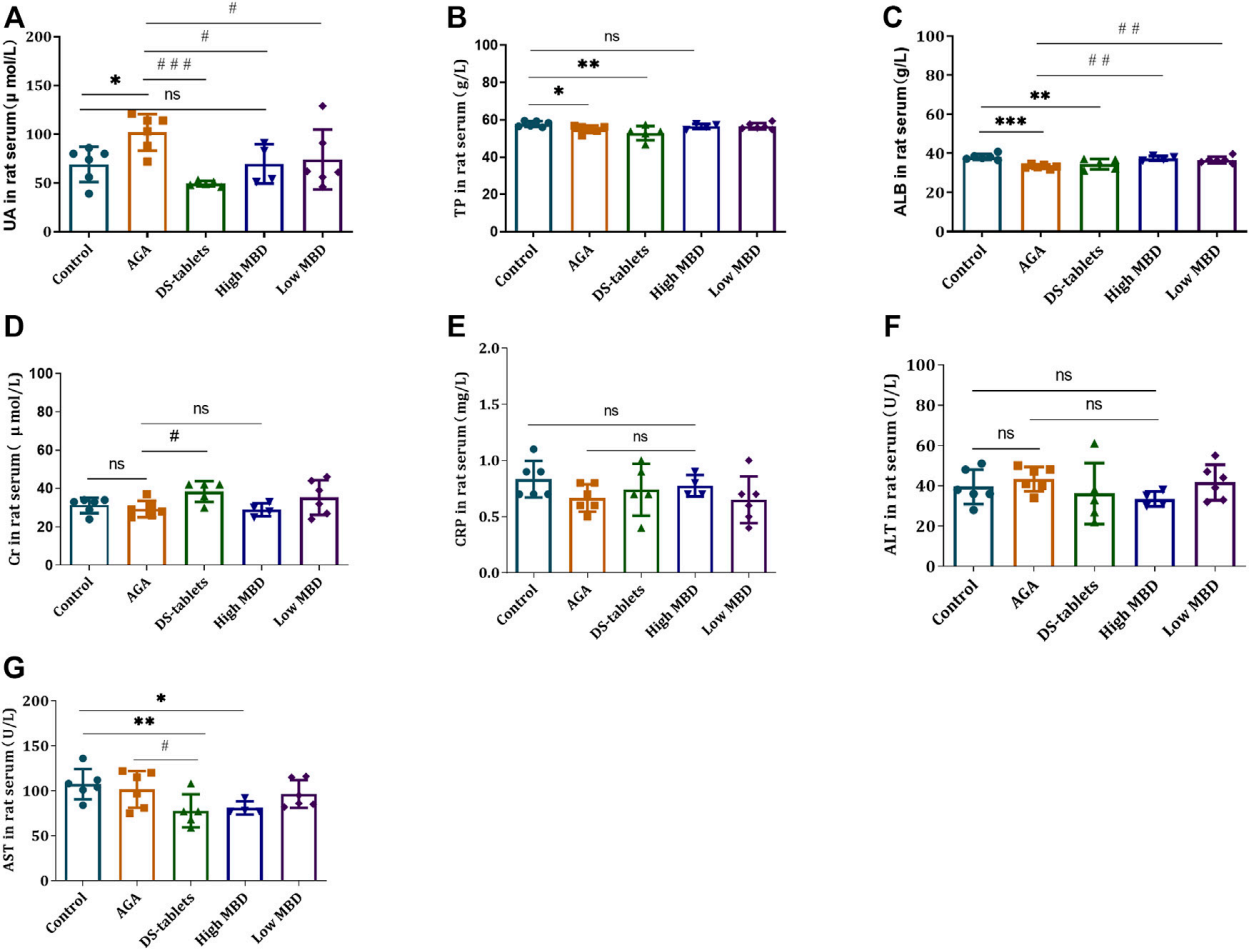
Effect of MBD on liver and kidney metabolic function and the expression of **(A)** uric acid (UA). **(B)** Total protein (TP). **(C)** Albumin (ALB). **(D)** Creatinine (Cr). **(E)** C-reactive protein (CRP). **(F)** Alanine aminotransferase (ALT). **(G)** Aspartate aminotransferase (AST) in serum (*n* = 6/group). ^*^ *p < 0.05,* ^* *^ *p* < 0.01, ^* * *^ *p < 0.001* indicated that the difference was statistically significant compared with the Control group; ^*#*^ *p < 0.05,* ^*# #*^ *p < 0.01,* ^# # #^ *p < 0.001* compared with the AGA group, the difference was statistically significant. “ns” represents not significant.

Changes in liver and kidney metabolic function are regarded as a factor in gout. MBD and diclofenac sodium tablets can significantly alleviate the symptoms of gouty arthritis in rats induced by UA sodium salt, and the efficacy of different doses of MBD in the treatment of gouty arthritis is similar. To further clarify the effect of MBD on the changes in liver and kidney metabolism in rats with gouty arthritis, biochemical analysis was performed on the serum of rats. Cr, UA, TP, ALB, CRP, AST and ALT levels were detected. UA levels were significantly higher in the AGA group than in the control group, and both MBD and DS tablets reduced UA levels compared to the AGA group (Figures 4A–C). Cr, CRP and ALT levels were not significantly different between groups (Figures 4D–F), and AST levels were significantly lower (Figure 4G). The results indicated that oral MBD could alleviate the gout symptoms caused by a high-fat and high-sugar diet and sodium urate injection and restore the changes in liver and kidney metabolism caused by gouty arthritis, and MBD did not cause liver and kidney damage.

### Effect of MBD on the expression of IL-1β, TNF-α and TGF-β1

**FIGURE 5:**
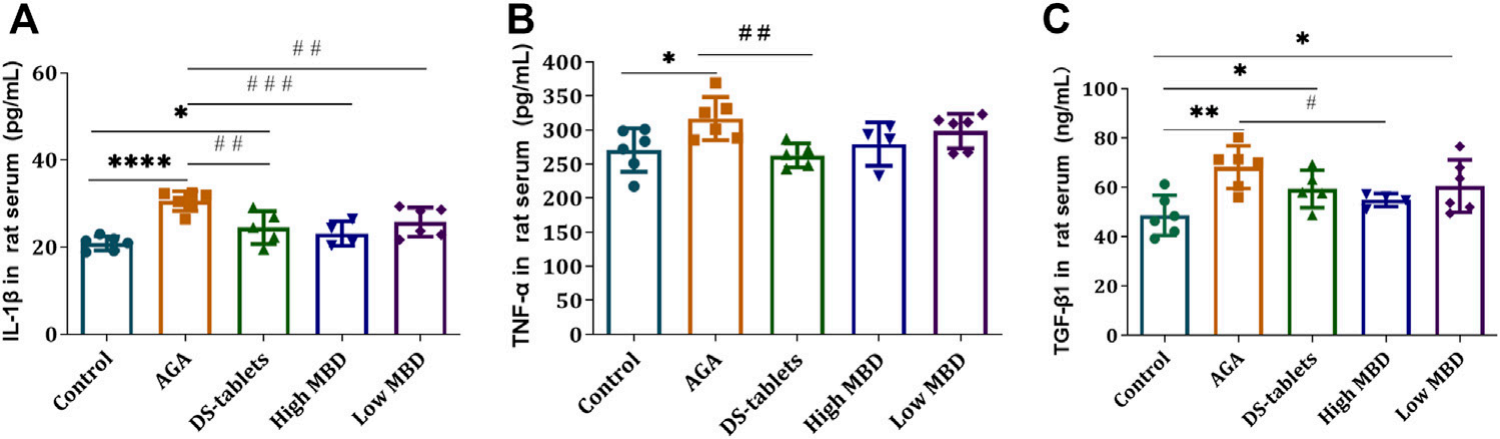
Effect of MBD on IL-1β, TNF-α and TGF-β1 expression levels in the serum of gouty arthritis rats. **(A)** IL-1β. **(B)** TNF-α. **(C)** TGF-β1. ^*^ *p < 0.05,* ^**^ *p < 0.01,* ^***^ *p < 0.001* indicate that the difference was statistically significant compared with the control group. ^*#*^ *p < 0.05,* ^##^ *p < 0.01,* ^###^ *p < 0.001* compared with the AGA group, the difference was statistically significant.

The levels of IL-1β, TNF-α and TGF-β1 in the serum of rats were used as indicators to evaluate the effect of MBD on gouty arthritis rats. Therefore, we studied the effect of MBD on the expression of IL-1β, TNF-α and TGF-β1. After injection of uric acid sodium salt into the ankle joint of rats, the levels of these three cytokines were detected by ELISA. The results showed that after the injection of sodium urate into the ankle joint of rats, the expression of IL-1β, TNF-α and TGF-β1 in the serum of rats was significantly increased compared with that in the blank group. Compared with the AGA group, the expression of IL-1β in the DS-tablet group and the high MBD group was significantly decreased (Figure 5A), and the expression of TNF-α in the DS-tablet group was also decreased, but there was no significant difference in the MBD group (Figure 5B). In addition, the expression of the TGF-β1 factor in the high MBD group was significantly reduced (Figure 5C). These results suggest that MBD can effectively inhibit the expression levels of these proinflammatory factors, thereby reducing the development of gouty arthritis.

### Effect of MBD on protein expression in synovial tissue of gouty arthritis rats

**FIGURE 6:**
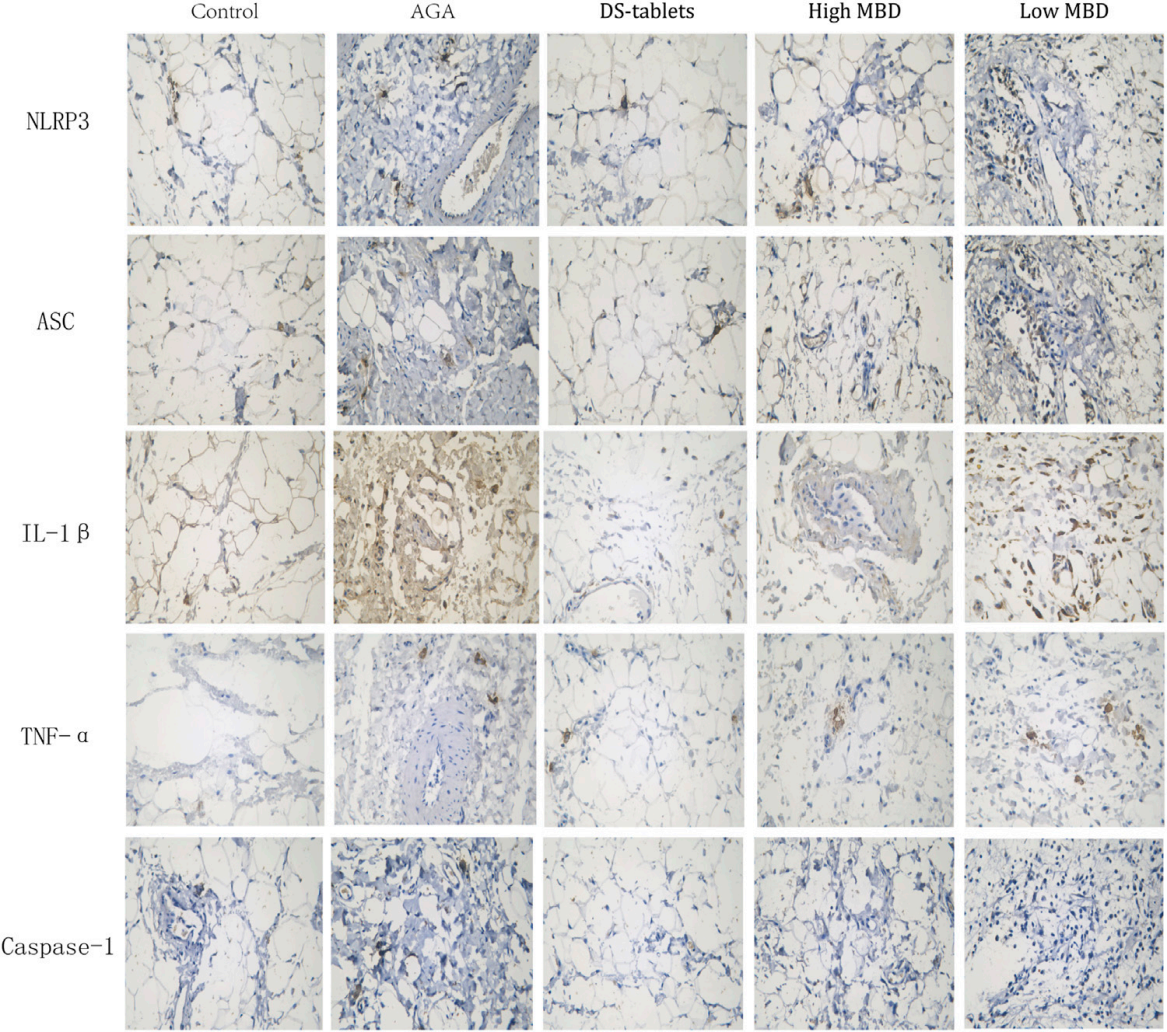
Expression of TNF-α, IL-1β, NLRP3, ASC and Caspase-1 in synovial tissue. Positive phase contrast microscope, magnification 400x.

The NLRP3 inflammasome is one of the chronic inflammatory pathways that regulate the body, and its components include NLRP3, ASC, and Caspase-1. NLRP3, the receptor, acts as an endogenous and exogenous risk signal, activates Caspase-I, upregulates IL-1β secretion and promotes the maturation and secretion of inflammatory cytokines. The expression of TNF-α, IL-1β, NLRP3, ASC, and Caspase-1 in synovial tissue was detected by the immunohistochemical SP method. From the experimental results, the expression of TNF-α, IL-1β, NLRP3, ASC, and Caspase-1 in the cytoplasm of synovial tissue was significantly upregulated in the AGA group compared with the control group. The results of the expression of TNF-α, IL-1β, NLRP3, ASC, and Caspase-1 in each treatment group compared to the AGA group indicated that the addition of MBD could effectively inhibit the expression of TNF-α, IL-1β, NLRP3, ASC and Caspase-1 proteins in the synovial tissue of rats with sodium urate-induced gouty arthritis (Figure 6).

### MBD regulates microbial community structure

**FIGURE 7:**
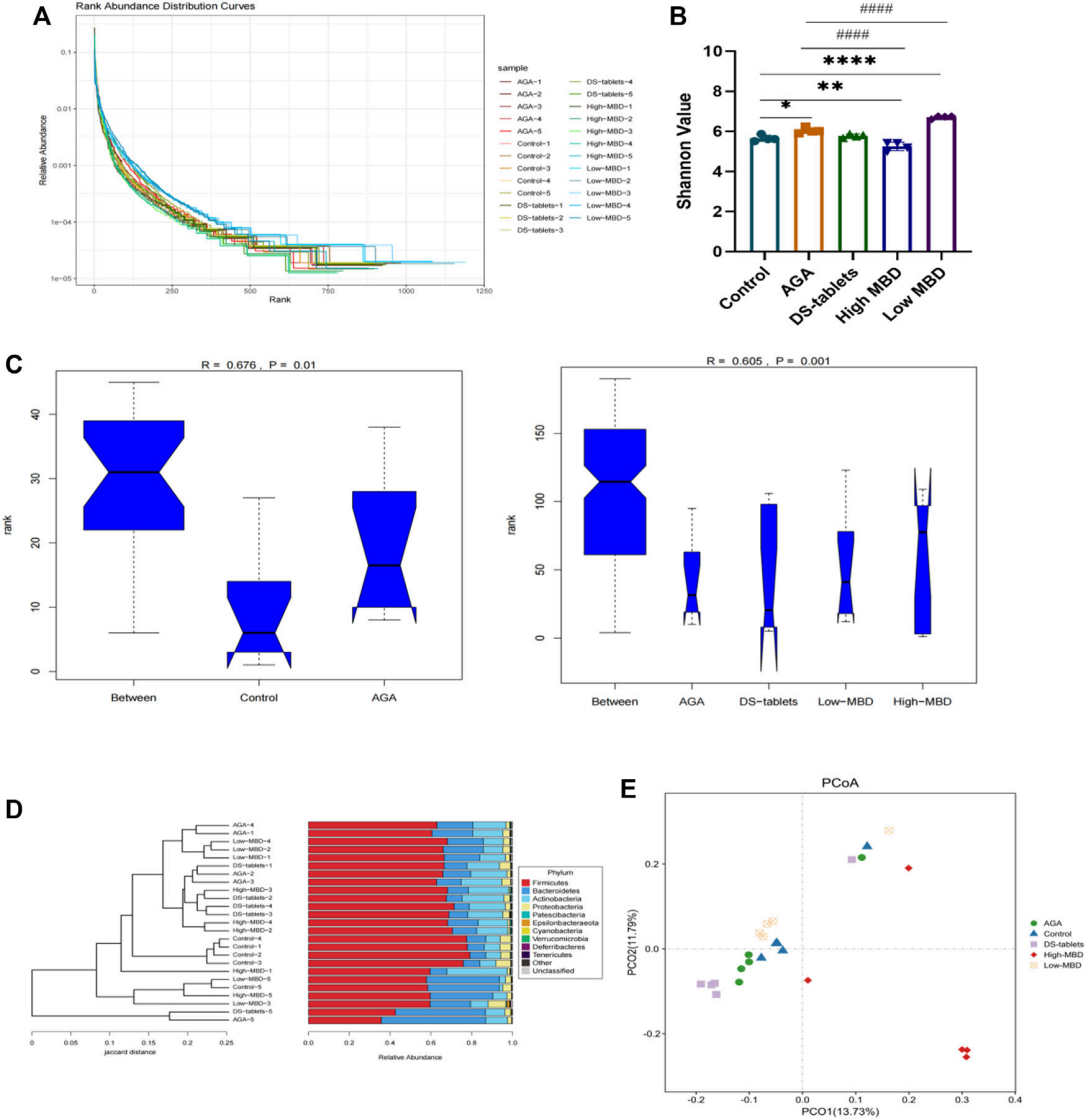
MBD regulated the microbial community structure. **(A)** Rank abundance curves provide a visual representation of the richness and uniformity of the taxa contained in the sample. **(B)** Effects of MBD on Shannon indices. **(C)** Analysis of similarity (ANOSIM) is a non-parametric test of the structure of microbial communities. The figure “between” is a visualization of the values of the distribution of distances between groups. Generally, *R > 0.75*: large differences; *R > 0.5*: moderate differences, *R > 0.25*: small differences. The confidence level of the statistical analysis is expressed as the *p*-value, with *p < 0.05* indicating statistical significance. **(D)** Hierarchical clustering analysis at the OTU level. **(E)** Based on data results from unweighted UniFrac between samples. ^*^ *p < 0.05,* ^**^ *p < 0.01,* ^***^ *p < 0.001* indicate that the difference was statistically significant compared with the control group. ^*#*^ *p < 0.05,* ^*##*^ *p < 0.01,* ^*###*^ *p < 0.001* compared with the AGA group, the difference was statistically significant.

Mounting evidence has proven that gut dysbiosis is a pivotal factor in acute gouty arthritis and that TCM can regulate the composition and function of gut microbiota. We next sequenced the bacterial V3-V4 region of the 16S rDNA gene to profile gut microbiota composition between normal and acute gouty arthritis in rats and evaluated the influence of MBD on the intestinal flora. A total of 2628101 tags were generated, with an average of 105124 tags per sample (Supplementary Figure S1). The rank abundance distribution curves of all samples tended to be flat, indicating that the amount of sequencing data was reasonable and the depth of sequencing was appropriate (Figure 7A). The alpha diversity indices for the Shannon index were significantly different among the various groups (Figure 7B). In addition, in conjunction with the ANOSIM grouping test, our analysis of the ranking results based on the unweighted UniFrac distances between samples showed that there were differences between the groups and that the statistical results were significantly different (Figure 7C). Both MBD and DS tablets could promote microbial richness and diversity. Hierarchical clustering analysis revealed that DS tablet-treated rats had a microbial community similar to that of acute gouty arthritis rats, while the microbiomes of MBD-treated rats were structured differently (Figure 7D). PCoA showed that samples could be separated based on the microbial community structure. The intragroup distance was smaller than the intergroup distance, showing that the microbiota composition of rats within the same group was almost the same as that of rats from a different group (Figure 7E). MBD can uniquely regulate the structure of the intestinal flora.

### Effect of MBD on the relative abundance of gut microbiota in rats with gouty arthritis

**FIGURE 8:**
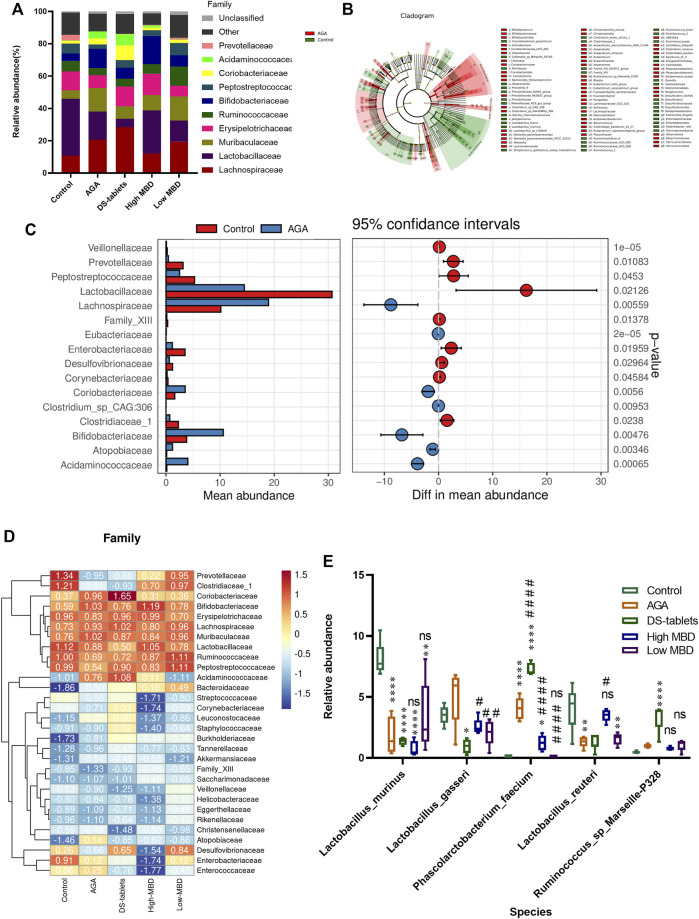
Effect of MBD on the relative abundance of various gut microbiota constituents in rats with gouty arthritis. **(A)** Differences in gut microbiota at the family level. **(B)** LEFSE analysis between the control group and the AGA group. **(C)** Differences at the genus level (phylum to species level, filtering for species whose sum of abundance in all samples was less than 0.1%) between the control and AGA groups were analysed using Welch’s *t* test, with a threshold of *p < 0.05* (or *0.01*) and a smaller *p*-value indicating a more significant difference. **(D)** Analysis of the correlation between characteristic intestinal flora and acute gouty arthritis symptoms. Positive correlations are displayed in red, and negative correlations are displayed in blue. The intensity of the colour is proportional to the correlation coefficient. **(E)** Relative abundance of characteristic intestinal flora after MBD and DS tablet treatment*.* ^*^ *p < 0.05,* ^* *^ *p < 0.01,* ^* * *^ *p < 0.001* indicated that the difference was statistically significant compared with the Control group; ^*#*^ *p < 0.05,* ^*# #*^ *p < 0.01,* ^# # #^ *p < 0.001* compared with the AGA group, the difference was statistically significant “ns” represents not significant.

Based on 16S rDNA gene sequencing, we observed that Lachnospiraceae, Lactobacillaceae, Muribaculaceae, Erysipelotrichaceae, Ruminococcaceae, Bifidobacteriaceae, Peptostreptococcaceae, and Prevotellaceae are the eight most common families. MBD restored the abundance of Lactobacillaceae, but DS tablets mainly altered the abundance of Lachnospiraceae, suggesting that these two drugs have different regulatory mechanisms (Figure 8A). LEFSE analysis showed that 58 bacterial genera were depleted in AGA rats, while 40 genera were enriched in these same samples (Figure 8B). It is worth noting that the abundance of bacteria capable of secreting short-chain fatty acids (SCFAs) was significantly reduced in the intestine of AGA rats (Figure 8B). Welch’s *t* test was used for the analysis of variance at the family level for the two subgroups. From the results, there were significant differences between the control and AGA groups for several genera of Lactobacillaceae*,* Lachnospiraceae, Prevotellaceae, and Bifidobacteriaceae (Figure 8C). A series of Spearman’s correlations were further conducted to elucidate the association between characteristic flora and symptoms of AGA. As shown in Figure 8D, compared to the control group, the abundance of Lachnospiraceae*,* Muribaculaceae and Bifidobacteriaceae increased in rats with AGA, while the abundance of Lactobacillaceae*,* Ruminococcaceae*,* Prevotellaceae and Peptostreptococcaceae showed a significant decrease in abundance (Figure 8D). Among them, Ruminococcaceae is the main microorganism that converts primary bile acids into secondary bile acids (the abundance of this group decreased in patients with ulcerative colitis), and Prevotellaceae is thought to be associated with the synthesis of SCFAs, the lack of which weakens their protective effect on the intestinal mucosal barrier and may lead to increased levels of enterogenic endotoxins. After treatment with MBD intervention, the disease-induced changes in flora abundance were restored (Figure 8E).

## Discussion

**FIGURE 9:**
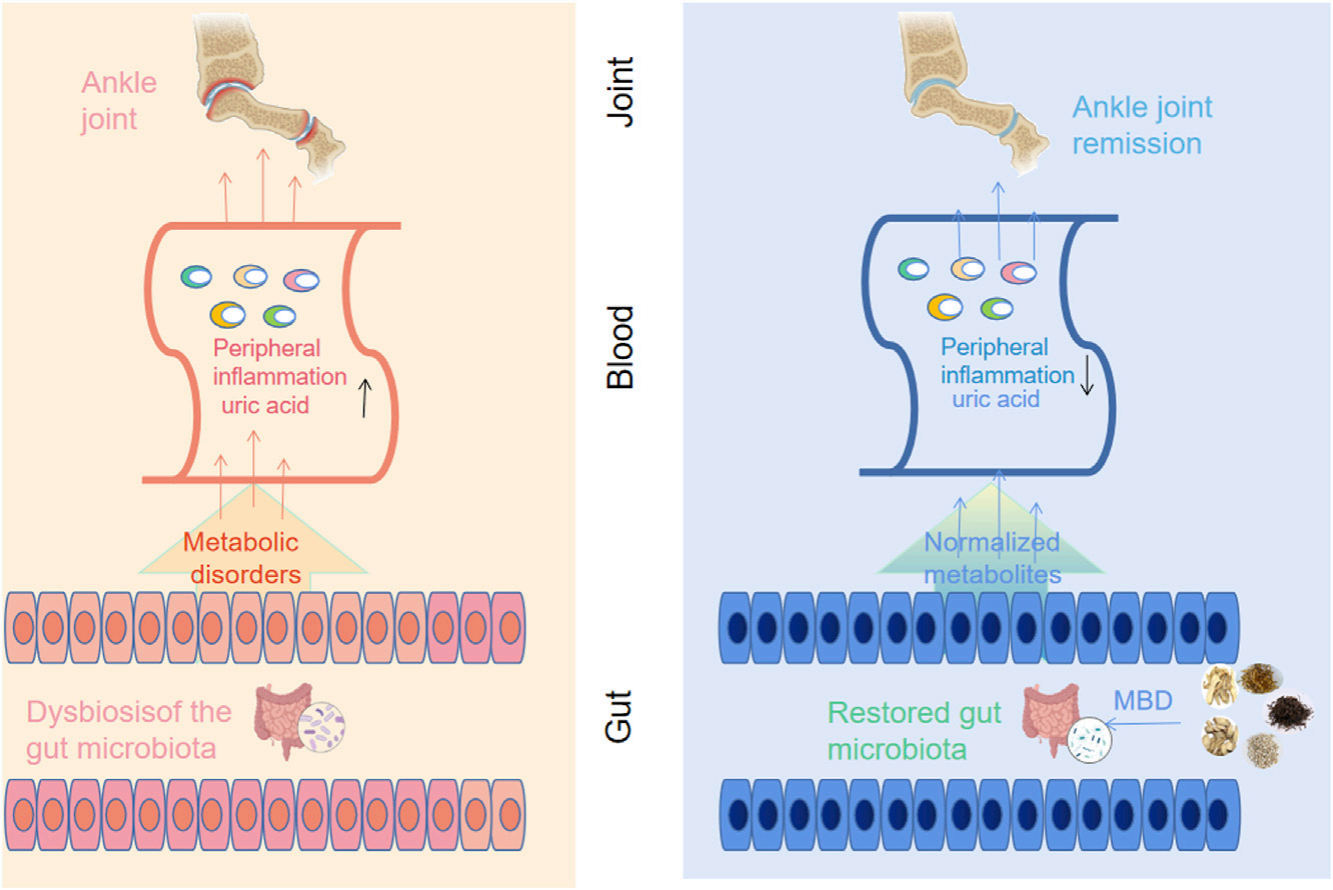
Correlations of arthritis with gut microbiota and UA metabolism. Along with the deposition of sodium urate in the joint cavity, intestinal bacterial disorders during the progression of AGA lead to abnormal uric acid metabolism and a rise in serum uric acid levels, accompanied by an inflammatory response (left panel). Oral administration of MBD with added flavour can restore the disorder of intestinal flora caused by AGA, normalize the metabolic disorder and reduce the peripheral inflammatory response, thus improving the symptoms of AGA (right panel).

The purine metabolism disease that causes gouty arthritis causes a rise in blood uric acid, the precipitation of urate crystals, and involvement of the joint capsule, joint synovium, cartilage bone, and surrounding connective tissue (Desai et al., 2017). People’s everyday lives are significantly impacted by the extreme pain brought on by acute attacks (Terkeltaub 2017). There has been much research in recent years that suggests there may be a link between gut flora and gouty arthritis (Chu et al., 2021). In the human body, the intestine has the largest microecological environment. Because it regulates host immunity and energy balance, the gut microbiota is thought of as an endocrine organ. Human health, particularly immunity and metabolism, is significantly impacted by gut microbiota imbalance (Kogut et al., 2020). In the context of increased serum uric acid levels, urate crystals precipitate and accumulate in the joint cavity, causing joint inflammation. However, changes in the gut microbiota during the progression of AGA lead to metabolic disturbances. The production of abnormal metabolites causes peripheral inflammation and exacerbates the symptoms of AGA (Figure 9). These insights into the pathogenesis of AGA may provide a new therapeutic solution by reorganizing the gut microbiota to favourably alleviate the symptoms of AGA.

New views and methods for comprehending and treating gouty arthritis have been developed as a result of the growing understanding of the part that gut flora plays in the condition (Henson 2021). According to studies, the Muribaculaceae, Ruminococcaceae, Lachnospiraceae, Lactobacillaceae, and Prevotellaceae families are the dominant flora in the rat intestine, with Muribaculaceae accounting for more than 20% of the total bacterial abundance. Additionally, research on the role of Prevotellaceae in human rheumatoid arthritis and arthritis in mouse models revealed that patients with rheumatoid arthritis had higher levels of Prevotellaceae in their intestines (Maeda et al., 2016). A different study, however, suggested that P. histicola might prevent the onset of arthritis (Marietta et al., 2016). These findings provide credence to the idea that different *Prevotella* species affect arthritis in various ways. In this work, MBD gavage was used to investigate the relationship between intestinal flora and a model of AGA caused by sodium urate. Intestinal flora and AGA have been linked, according to Xiaoying Lin et al. They used serum proinflammatory factors and intestinal ecology to explain how Simiao Decoction works to treat AGA (Lin et al., 2020). By employing a computer study of 16S rRNA gene amplicon sequence data in stool samples from gout patients and healthy controls, Michael A. Henson further demonstrated that the gut flora is disrupted in gout (Henson 2021). Furthermore, Nobuhito Nemoto et al. showed that the immunological and gut flora distribution varied between the acute and recurring phases of arthritis, with significant alterations in gut immunity and the environment as arthritis advanced (Nemoto et al., 2020). In conclusion, the intestinal flora modulates the development of AGA induced by sodium urate, but more research is required to pinpoint the precise mechanism underlying this relationship.

Numerous randomized trials have shown that Baihu Jia Guizhi Decoction, which is based on the traditional prescription Baihu Decoction, is a potent medication for the treatment of joint disease and can reduce rheumatoid arthritis inflammation by modulating the succinate SUCNR1 metabolic signalling pathway (Chen et al., 2019). Although the anti-inflammatory properties of Baihu Decoction have also been linked to the treatment of sepsis and systemic inflammatory response syndrome by lowering blood glucose levels, nothing is known about its anti-inflammatory effects on AGA, and the mechanism of action is still unclear (Yang and Hou 2010). In our work, MBD substantially reduced sodium urate-induced rat toe oedema without discernible adverse effects after just 21 days. The addition of MBD also reduced the expression of IL-1β and TGF-β1 in serum, alleviated synovial inflammation (NLRP3, TNF-α, IL-1β, NLRP3, ASC, Caspase-1), and restored the abundance of bacteria caused by disease.

An increasing number of studies have recently examined how herbal medicines and gut microbes interact. Chinese herbal remedies that are taken orally have the ability to change the makeup of intestinal microbes, control their metabolism, and transform herbal remedies into herbal compounds after digestion (Feng et al., 2019). This interaction results in the production of herbal substances modified by intestinal bacteria and intestinal microbial metabolites. In this study, we found a strong association between gut microbiota imbalance (i.e., dysbiosis) and AGA development. The gut serves as a dominant organ for UA distribution, a potential target for UA suppression and a key pathway for extrarenal UA elimination (Yun et al., 2017). Bacteria in the gut are able to decompose UA to allantoin (Vieira et al., 2015). Despite the limited understanding of the mechanism underlying the interaction between the gut microbiota and UA metabolism, the gut microbiota has been widely recognized to play a crucial role in UA excretion. A study reported a reduced blood uric acid (BUA) level in *Bacteroides fragilis* (BF) gnotobiotic mice (Hsu et al., 2015). *Lactobacillus* PA3 in the gut helps prevent and mitigate gout by reducing the UA level *via* the purine metabolism pathway. Another study revealed that the UA level in bacteria-containing fat bodies increased by 20 times when the symbiotic bacteria in these bodies were killed by antibiotics, which might be attributed to UA recycling by *Blattabacteria* (Maeda et al., 2016). With the development of the global economy, the Chinese population has somewhat been affected by the purine-heavy, high-fat Western diet, which presents a real challenge to the gut microbiota and genomes. In an organism with low purine intake, the gut microbiota can recycle urea and uric acid to synthesize essential amino acids for the host (Zhu et al., 2018). Therefore, the serum UA level depends heavily on the gut’s ability to metabolize UA (Lim et al., 2014). As an independent marker or risk factor, UA is the end-product of endogenous purine metabolism, of which the conversion suggests the involvement of a series of enzymes that may lead to abnormal UA metabolism (Pan et al., 2020). UA production and excretion are two key players in its metabolism, while certain endogenous microorganisms have been found to play a specific role in UA metabolism (Mendez-Salazar et al., 2021). Compared with healthy individuals, hyperuricaemia (HUA) (Vieira et al., 2015) and gout patients experience differential changes in the gut microbiota (Szulinska et al., 2018). The gut microbiota composition is altered following medical treatment to reduce UA, while probiotics produce an antagonistic effect on abnormal UA metabolism (Yun et al., 2017; Yu et al., 2018; Mendez-Salazar et al., 2021).

In conclusion, animal studies and clinical trials have shown that gut flora can prevent and treat autoimmune disorders by modifying immune function. It has been proposed that examining gout patients’ gut flora rather than only measuring serum uric acid levels may help predict illness earlier and more precisely (Guo et al., 2016). There will be a greater understanding of the mechanisms by which gut flora regulate immune function with the development of bacteraemia, metabolomics, and genomics, but the complexity of their interactions with the human body due to the abundance and diversity of gut flora, the various metabolites of particular gut microbes, and the interactions between different species of gut flora suggest the need to study not only the different species of gut flora but also to concentrate on their interactions. This emphasizes the necessity to research not only the various gut flora but also how the disease is influenced by the balance of these bacteria. Even so, further research is necessary to fully understand the effects of gut microbiota on the immune system in gout and which gut bacteria affect urate breakdown in the host. The available results do, however, point in a direction for future research: by examining the gut microbiota distribution characteristics in gout patients, the ecological balance can be restored through targeted gut microbiota manipulation for the ultimate goal of treating the condition (An et al., 2019).

## Data Availability

The datasets presented in this study can be found in online repositories. The names of the repository/repositories and accession number(s) can be found in the article/Supplementary Material.
