# Supplementary figures and images for: Modified Baihu decoction therapeutically remodels gut microbiota to inhibit acute gouty arthritis
Source: Front Physiol. 2022 Dec 15;13:1023453. doi: 10.3389/fphys.2022.1023453 (PMC9798006; doi:10.3389/fphys.2022.1023453)

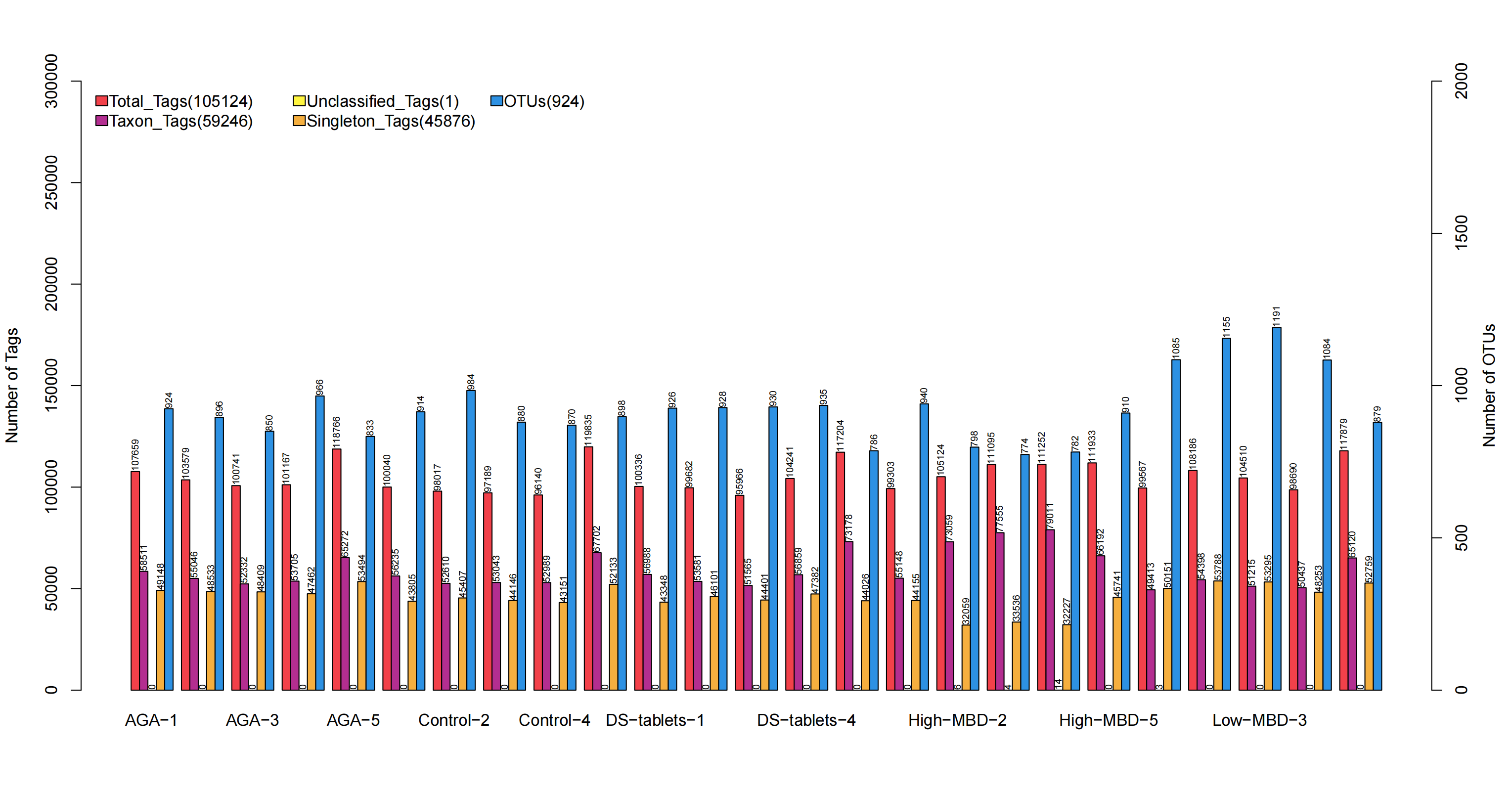

Supplement: Supplementary file 1 [file Image2.TIF]

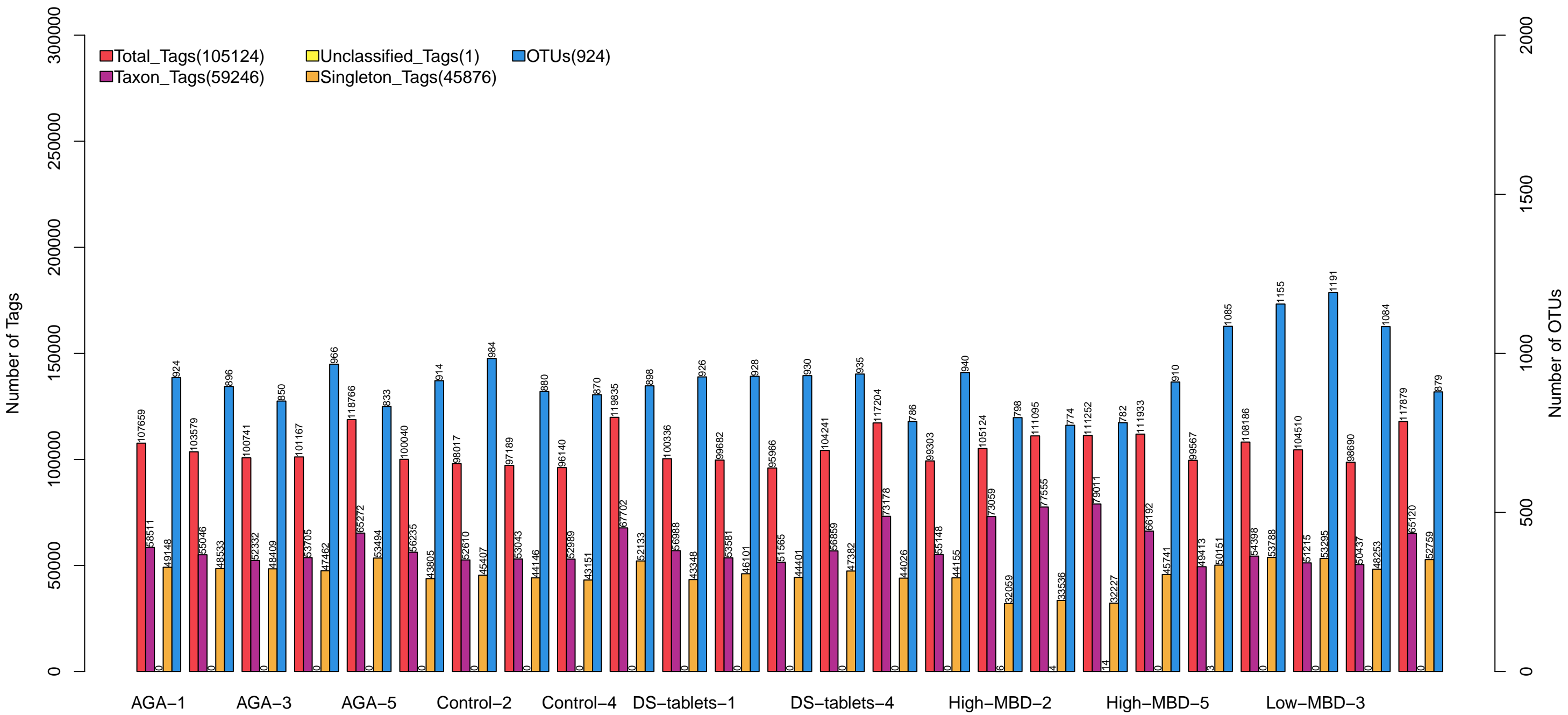

Supplement: Supplementary file 2 [file DataSheet1.PDF]

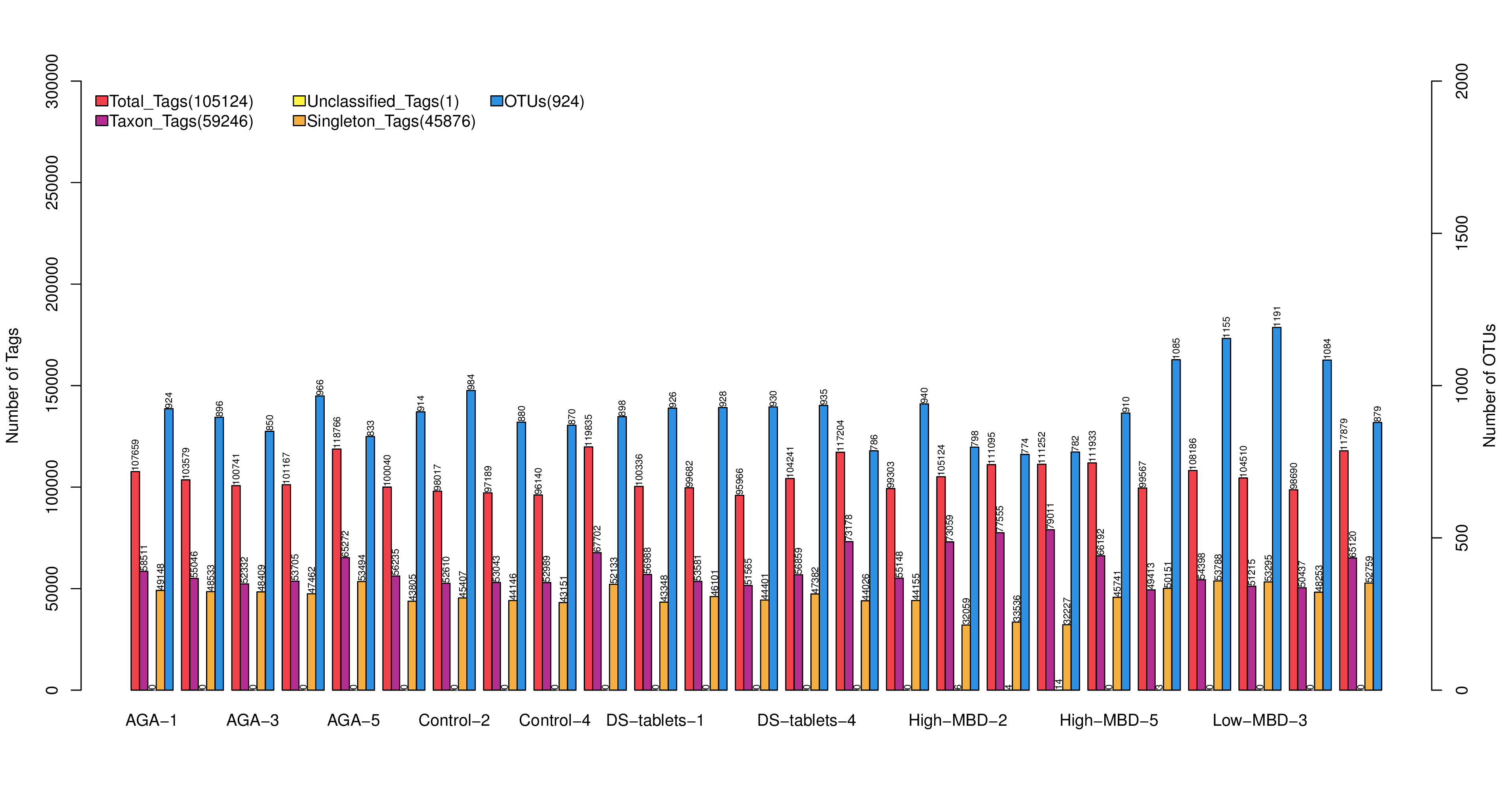

Supplement: Supplementary file 3 [file Image1.PNG]
